# The E3 ubiquitin ligases β-TrCP and FBXW7 cooperatively mediates GSK3-dependent Mcl-1 degradation induced by the Akt inhibitor API-1, resulting in apoptosis

**DOI:** 10.1186/1476-4598-12-146

**Published:** 2013-11-22

**Authors:** Hui Ren, Junghui Koo, Baoxiang Guan, Ping Yue, Xingming Deng, Mingwei Chen, Fadlo R Khuri, Shi-Yong Sun

**Affiliations:** 1 Department of Respiration, First Affiliated Hospital of Medical College of Xi’an Jiaotong University, Xi’an, Shaanxi, P R China; 2 Department of Hematology and Medical Oncology, Emory University School of Medicine and Winship Cancer Institute, Atlanta, Georgia, USA; 3 Department of Radiation Oncology, Emory University School of Medicine and Winship Cancer Institute, Atlanta, Georgia, USA

**Keywords:** API-1, GSK3, Mcl-1, E3 ubiquitin ligase, Apoptosis, Lung cancer

## Abstract

**Background:**

The novel Akt inhibitor, API-1, induces apoptosis through undefined mechanisms. The current study focuses on revealing the mechanisms by which API-1 induces apoptosis.

**Results:**

API-1 rapidly and potently reduced the levels of Mcl-1 primarily in API-1-senstive lung cancer cell lines. Ectopic expression of Mcl-1 protected cells from induction of apoptosis by API-1. API-1 treatment decreased the half-life of Mcl-1, whereas inhibition of the proteasome with MG132 rescued Mcl-1 reduction induced by API-1. API-1 decreased Mcl-1 levels accompanied with a rapid increase in Mcl-1 phosphorylation (S159/T163). Moreover, inhibition of GSK3 inhibited Mcl-1 phosphorylation and reduction induced by API-1 and antagonized the effect of API-1 on induction of apoptosis. Knockdown of either FBXW7 or β-TrCP alone, both of which are E3 ubiquitin ligases involved in Mcl-1 degradation, only partially rescued Mcl-1 reduction induced by API-1. However, double knockdown of both E3 ubiquitin ligases enhanced the rescue of API-1-induced Mcl-1 reduction.

**Conclusions:**

API-1 induces GSK3-dependent, β-TrCP- and FBXW7-mediated Mcl-1 degradation, resulting in induction of apoptosis.

## Background

API-1 (4-amino-5,8-dihydro-5-oxo-8-β-D-ribofuranosyl-pyrido[2,3-*d*]pyrimidine-6-carboxamide) is a recently identified novel Akt inhibitor. It inhibits Akt activity through binding to the pleckstrin homology domain of Akt and blocking its membrane translocation [1]. API-1 possesses promising anticancer activity, evidenced by its ability to suppress cell growth, induce apoptosis and inhibit the growth of cancer xenografts, particularly those with activated Akt, in nude mice [1]. We have recently shown that API-1 facilitates c-FLIP degradation, induces apoptosis and enhances tumor necrosis factor-related apoptosis-inducing ligand (TRAIL)-induced apoptosis in human non-small cell lung cancer (NSCLC) cells [2]. c-FLIP degradation clearly contributes to the enhancement of TRAIL-induced apoptosis by API-1 [2]. However, the mechanisms by which API-1 induces apoptosis in cancer cells and the additional mechanisms accounting for API-1-mediated augmentation of TRAIL-induced apoptosis are largely unknown.

In addition to the extrinsic death receptor-mediated apoptotic pathway, which is characterized by the oligomerization of cell surface death receptors and activation of caspase-8, the intrinsic apoptotic pathway that involves the disruption of mitochondrial membranes, release of cytochrome c and activation of caspase-9 is another critical apoptotic mechanism [3]. It is known that the intrinsic apoptotic pathway is negatively regulated by anti-apoptotic Bcl-2 family members (e.g., Mcl-1, Bcl-2 and Bcl-X_L_) and inhibitor of apoptosis proteins (IAPs; e.g., survivin). In general, downregulation of these anti-apoptotic proteins can trigger apoptosis or augment TRAIL-induced apoptosis [4-6].

Among the anti-apoptotic Bcl-2 family members, Mcl-1 is known to be a short-lived protein that undergoes ubiquitination/proteasome-mediated degradation [7]. One degradation mechanism involves glycogen synthase kinase 3 (GSK3), which phosphorylates Mcl-1 at S159, triggering Mcl-1 degradation [8,9]. It has been suggested that Mcl-1 phosphorylation at S159 facilitates the association of Mcl-1 with the E3 ligase β-transducin repeats-containing protein (β-TrCP), resulting in β-TrCP-mediated ubiquitination and degradation of Mcl-1. Recently two studies have suggested that phosphorylation at S159 enhances the association of Mcl-1 with the E3 ligase F-box/WD repeat-containing protein 7 (FBXW7), resulting in FBXW7-mediated ubiquitination and degradation of Mcl-1 [10,11].

In this study, we focused on revealing mechanisms by which API-1 induces apoptosis of cancer cells and uncovered GSK3-dependent Mcl-1 degradation as a critical mechanism accounting for induction of apoptosis by API-1. This mechanism also contributes to augmentation of TRAIL-induced apoptosis by API-1.

## Methods

### Reagents

API-1 (NSC177233) was obtained from the National Cancer Institute (Bethesda, MD). MK2206 was purchased from Active Biochem (Maplewood, NJ). They were dissolved in DMSO and stored at -80°C. Soluble recombinant human TRAIL was purchased from PeproTech, Inc. (Rocky Hill, NJ). The proteasome inhibitor MG132, the protein synthesis inhibitor cycloheximide (CHX) and the GSK3 inhibitor SB216763 were purchased from Sigma Chemical Co. (St. Louis, MO). The neddylation inhibitor MLN4924 was provided by Millennium Pharmaceuticals, Inc (Cambridge, MA). Expression plasmids in pCI vector carrying wild-type and mutant (S159A) human Mcl-1 were provided by Dr. X. Deng (Emory University, Atlanta, GA). Mouse monoclonal survivin and caspase-8 antibodies and rabbit polyclonal Bim, caspase-9 and PARP antibodies were purchased from Cell Signaling Technology (Danvers, MA). Mouse monoclonal caspase-3 antibody was purchased from Imgenex (San Diego, CA). Rabbit polyclonal Mcl-1, Bad, Bcl-X_L_ and SKP1 and mouse monoclonal Bcl-2, Cul-1 and α-tubulin antibodies were purchased from Santa Cruz Biotechnology, Inc. (Santa Cruz, CA). GSK3α/β antibody was purchased from Upstate/Millipore (Billerica, MA). Mouse monoclonal Bax and rabbit polyclonal glyceraldehyde 3-phosphate dehydrogenase (GAPDH) antibodies were purchased from Trevigen Inc. (Gaithersburg, MD). Both polyclonal and monoclonal actin antibodies were purchased from Sigma Chemical Co.

### Cell lines and cell culture

The human NSCLC cell lines used in this study including those stably expressing ectopic Mcl-1 or survivin were described previously [12-14]. A549 cells were recently authenticated by Genetica DNA Laboratories, Inc. (Cincinnati, OH) through analyzing short tandem repeat DNA profile; other cell lines have not been authenticated. HCT116/wild type (WT) and HCT116/FBXW7-KO cell lines were kindly provided by Dr. B. Vogelstein (Johns Hopkins University School of Medicine, Baltimore, MA). These cell lines were grown in monolayer culture in RPMI 1640 medium or McCoy’s medium supplemented with glutamine and 5% fetal bovine serum at 37°C in a humidified atmosphere consisting of 5% CO_2_ and 95% air.

### Cell survival and apoptotic assays

Cells were seeded in 96-well cell culture plates and treated the next day with the given agents. Viable cell numbers were determined using sulforhodamine B (SRB) assay as described previously [15]. Combination index (CI) for drug interaction (e.g., synergy) was calculated using the CompuSyn software (ComboSyn, Inc.; Paramus, NJ). Apoptosis was evaluated with an annexin V-PE apoptosis detection kit (BD Biosciences; San Jose, CA) according to the manufacturer’s instructions. We also detected caspases and PARP cleavage by Western blot analysis as described below as additional indicators of apoptosis.

### Western blot analysis

Preparation of whole-cell protein lysates and Western blot analysis were described previously [16,17].

### Small interfering RNA (siRNA) and transfection

GSK3α/β siRNA (#6301) was purchased from Cell Signaling Technology. FBXW7 siRNA that targets the sequence of 5′-AACACAAAGCTGGTGTGTGCA-3′ [18] was synthesized from Qiagen (Valencia, CA) and used in our previous study [13]. Cullin 1 (Cul1; sc-35126), SKP1 (sc-29482) and β-TrCP (sc-37178) siRNAs were purchased from Santa Cruz Biotechnology, Inc. siRNA transfection was performed with HiPerFect transfection reagent (Qiagen) or Lipofectamine 2000 (Invitrogen) following the manufacturer’s instructions.

### Reverse transcription-PCR (RT-PCR)

To confirm knockdown efficiencies of β-TrCP and FBXW7 siRNA, Control, β-TrCP, FBXW7 and β-TrCP plus FBXW7 siRNAs were transfected into H1299 cells with Lipofectamine 2000. After 48 h, total RNA was then prepared from the cells by Trizol (Sigma). Reverse transcription was performed with iScript select cDNA synthesis kit (Bio-Rad; Hercules, CA), followed with PCR using primers as follows: β-TrCP, 5′-CCCCAACTGACATTACCC-3′ (forward) and 5′-TCGAATACAACGCACCAA-3′ (reverse) [19]; FBXW7, 5′-AAAGAGTTGTTAGCGGTTCTCG-3′ (forward) and 5′-CCACATGGATACCATC AAACTG-3′ (reverse) [20]; GAPDH, 5′-TGATGACATCAAGAAGGTGGTGAAG-3′ (forward) and 5′-TCCTTGGAGGCCATGTGGGCCAT-3′ (reverse) [21]. Using the same assay, Mcl-l mRNA expression in cells exposed to API-1 was detected with the following primers: 5′-TAAGGACAAAACGGGACTGG-3′ (forward) and 5′-ACCAGCTCCTACTCCAGCAA-3′ (reverse) [22].

## Results

### API-1 induces Mcl-1 reduction in API-1 sensitive NSCLC cell lines

**Figure 1:**
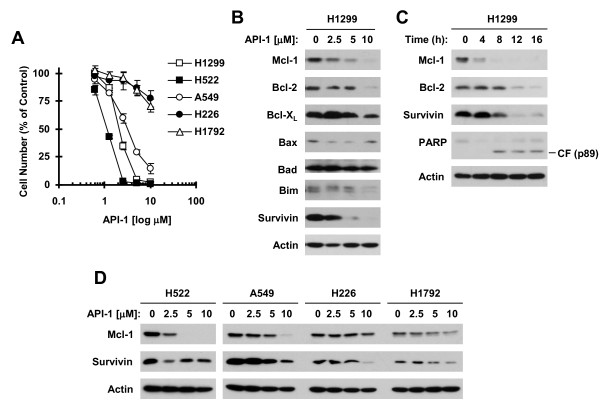
**API-1 decreases Mcl-1 levels (B-D) in API-1-sensitive NSCLC cell lines (A). A**, The given cell lines were treated with different concentrations of API-1 ranging from 10 to 0.5 μM for 3 days. Cell numbers were then estimated with the SRB assay. Data are means ± SDs of four replicate determinations. **B**-**D**, The given cell lines were treated with different concentrations of API-1 as indicated for 12 h **(B and D)** or 5 μM API-1 for the indicated times **(C)**. The cells were then harvested for preparation of whole-cell protein lysates and subsequent Western blot analysis to detect the indicated proteins.

Human NSCLC cell lines exhibited varied sensitivities to API-1 as evaluated with the SRB assay after a 3-day incubation (Figure 1A). Among them, H1299, H522 and A549 were sensitive to API-1, whereas H226 and H1792 were quite resistant to API-1. Since our previous work investigated the effects of API-1 on the expression of several proteins (e.g., c-FLIP, DR4 and DR5) involved in the extrinsic apoptotic pathway [2], we focused our current study on determining the effects of API-1 on modulation of the levels of several proteins (e.g., Mcl-1 and survivin) involved in regulation of the intrinsic apoptotic pathway. In H1299 cells, API-1 decreased the levels of Mcl-1 and survivin at even 2.5 μM and the levels of Bcl-2 and Bcl-X_L_ at 10 μM with no apparent increase in the levels of the pro-apoptotic proteins, Bax, Bad and Bim (Figure 1B). Moreover, we found that API-1 decreased Mcl-1 levels at 4 h, survivin levels at 8 h and Bcl-2 levels at 12 h post treatment (Figure 1C), indicating that Mcl-1 reduction is a rapid event ahead of survivin and Bcl-2 decrease in the process of API-1-induced apoptosis. By further comparing effects of API-1 on reducing Mcl-1 and survivin in 4 more NSCLC cell lines with different sensitivities to API-1, We found that API-1 reduced Mcl-1 levels effectively in H522 and A549 cells, which are sensitive to API-1, but only minimally in H226 and H1792 cells, which are insensitive to API-1. We detected survivin reduction in all four cell lines post API-1 treatment regardless of their sensitivities to API-1 (Figure 1D). These data emphasize the relevance and importance of Mcl-1 reduction in cell response to API-1.

### Enforced expression of ectopic Mcl-1 protects NSCLC cells from induction of apoptosis by API-1 or API-1 combined with TRAIL

**Figure 2:**
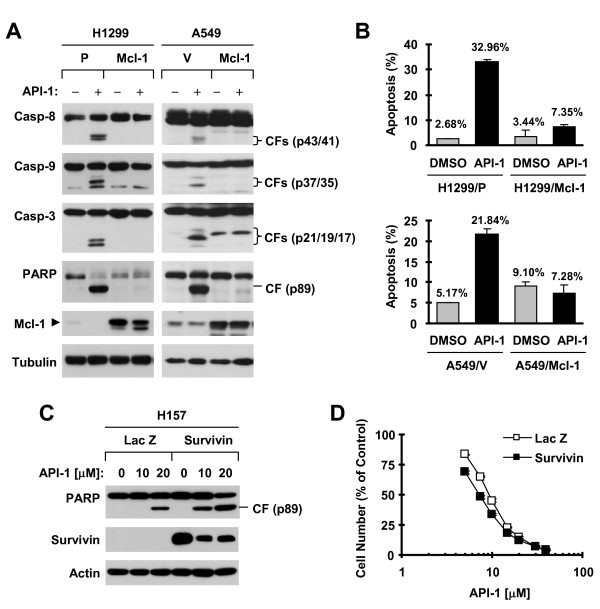
**Enforced expression of ectopic Mcl-1 (A and B), but not survivin (C and D), protects cells from API-1-induced apoptosis including caspase activation. A** and **B**, The indicated stable transfectants were exposed to 5 μM API-1 for 24 h and then harvested for preparation of whole-cell protein lysates and subsequent Western blot analysis to detect caspase cleavage and Mcl-1 expression **(A)** and for detection of apoptosis (i.e., annexin V-positive cells) with flow cytometry **(B)**. P, parental; V, vector. **C**, The indicated cell lines were exposed to the given concentrations of API-1 for 24 h and then harvested for preparation of whole cell protein lysates and subsequent Western blot analysis. **D**, The indicated H157 stable transfectants were treated with different concentrations of API-1 as indicated for 3 days. Cell numbers were estimated with SRB assay. The data are means ± SDs of four replicate determinations. CF, cleaved fragment.

We next determined whether Mcl-1 and survivin reduction is indeed involved in mediating induction of apoptosis by API-1. To this end, we enforced the expression of ectopic Mcl-1 or survivin in NSCLC cells and then examined their protective effects on API-1-induced apoptosis. In both H1299 and A549 cells, API-1 treatment caused clear cleavage of caspase-8, caspase-9, caspase-3 and PARP in vector control cell lines; these effects were not observed or were drastically diminished in Mcl-1-transfected cell lines (Figure 2A). Moreover, API-1 did not or only minimally increased annexin V-positive (i.e., apoptotic) cell populations in both H1299/Mcl-1 and A549/Mcl-1 cell lines, but drastically in their matched control counterparts (Figure 2B). These results clearly indicate that enforced expression of ectopic Mcl-1 protects cells from undergoing API-1-induced apoptosis, suggesting that Mcl-1 reduction is indeed critical for mediating apoptosis induced by API-1. In contrast, enforced expression of ectopic survivin failed to protect cells from killing by API-1: survivin-transfected cells were even more sensitive than the control Lac Z-expressing cells to the effects of API-1 on PARP cleavage and cell survival (Figure 2C-D), suggesting that survivin reduction is less important than Mcl-1 in mediating induction of apoptosis by API-1.

**Figure 3:**
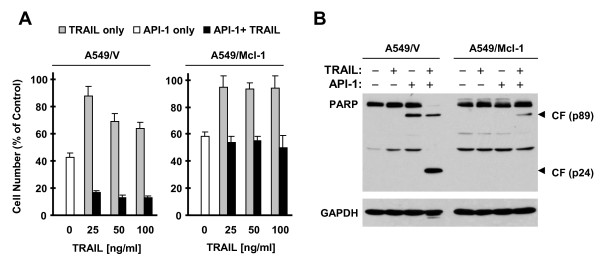
**Figure Enforced expression of ectopic Mcl-1 in A549 cells protects cells from apoptosis induced by the API-1 and TRAIL combination. A**, The indicated stable transfectants seeded in 96 well-plates were treated with 5 μM API-1 alone, the indicated concentrations of TRAIL alone and API-1 in combination with TRAIL. After 24 h, the cell numbers were estimated with SRB assay. The data are means ± SDs of four replicate determinations. **B**, The indicated stable transfectants were treated with 5 μM API-1 alone, 30 ng/ml TRAIL alone or their combination for 22 h and then harvested for preparation of whole-cell protein lysates and subsequent Western blot analysis to detect PARP cleavage. CF, cleaved form.

We also asked whether Mcl-1 reduction contributes to enhancement of TRAIL-induced apoptosis by API-1. We found that the combination of API-1 and TRAIL was far more active than either agent alone in decreasing survival and inducing PARP cleavage in A549/V cells as we previously reported [2]; these effects were substantially diminished in A549/Mcl-1 cells (Figure 3). Hence, it is clear that enforced Mcl-1 expression protects cells from undergoing apoptosis by the API-1 and TRAIL combination, suggesting that Mcl-1 reduction also contributes to augmentation of TRAIL-induced apoptosis by API-1. Collectively, these results provide a strong justification to focus our further studies on addressing the mechanisms by which API-1 induces Mcl-1 reduction.

### API-1 facilitates proteasomal degradation of Mcl-1

**Figure 4:**
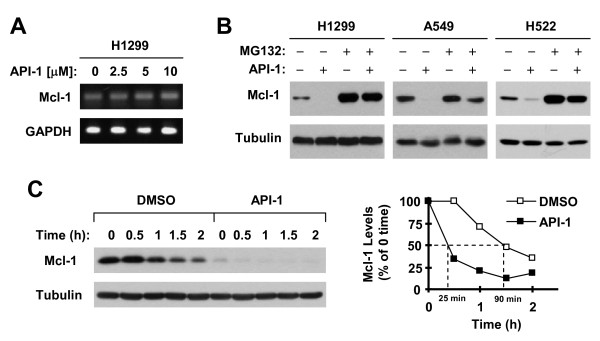
**API-1 induces proteasome-mediated Mcl-1 degradation (B) and decreases Mcl-1 stability (C) without altering Mcl-1 mRNA levels (A). A**, H1299 cells were exposed to the indicated concentrations of API-1 for 8 h. Total cellular RNA was then isolated from the cells for detection of Mcl-1 and GAPDH (internal control) mRNAs with RT-PCR. **B**, The indicated cell lines were pre-treated with 20 μM MG132 for 30 minutes prior to the addition of 5 μM API-1. After co-treatment for an additional 4 h, the cells were harvested for preparation of whole-cell protein lysates and subsequent Western blot analysis. **C**, H1299 cells were treated with 5 μM API-1 for 8 h. The cells were then washed with PBS 3 times and refed with fresh medium containing 10 μg/ml CHX. At the indicated times, the cells were harvested for preparation of whole-cell protein lysates and subsequent Western blot analysis. Protein levels were quantified with NIH Image J software (Bethesda, MA) and were normalized to actin. The results were plotted as the relative Mcl-1 levels compared to those at the time 0 of CHX treatment (right panel).

To elucidate the mechanisms by which API-1 reduces Mcl-1 levels, we detected Mcl-1 mRNA expression in cells exposed to different concentrations of API-1 with RT-PCR and found that API-1 did not alter Mcl-1 mRNA levels (Figure 4A). Since Mcl-1 is known to be a protein subject to proteasomal degradation [23,24], we next determined whether API-1-induced Mcl-1 reduction is due to enhancement of its degradation. Hence, we compared the effects of API-1 on Mcl-1 reduction in the absence and presence of the proteasome inhibitor MG132 in a few of NSCLC cell lines. API-1 effectively decreased Mcl-1 levels in the absence of MG132, but failed to do so in the presence of MG132 (Figure 4B), indicating that API-1 indeed induces proteasomal degradation of Mcl-1. Moreover, we found that the half-life of Mcl-1 in API-1-treated cells (about 20 min) was shorter than that in DMSO-treated cells (about 90 min) (Figure 4C), indicating that API-1 decreases the stability of Mcl-1. Taken together, we conclude that API-1 facilitates proteasomal degradation of Mcl-1, resulting in Mcl-1 reduction.

### API-1 induces GSK3-dependent Mcl-1 degradation

**Figure 5:**
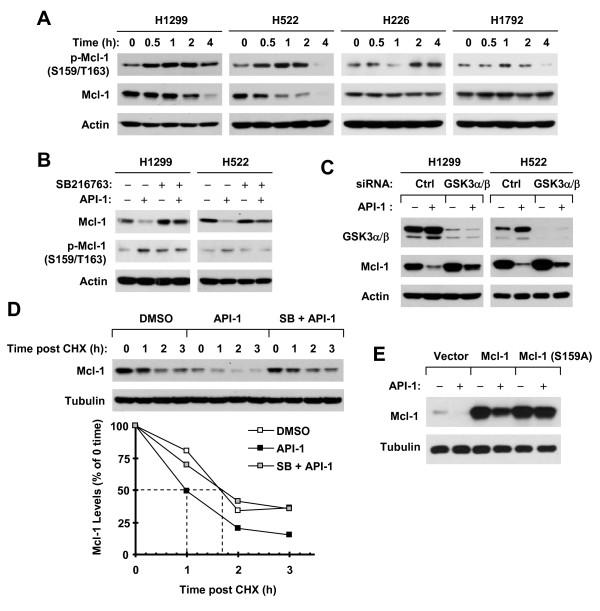
**API-1 induces GSK3-dependent Mcl-1 phosphorylation and degradation. A**, The given cell lines were treated with 5 μM API-1 for different times as indicated. **B**, The given cell lines were pre-treated with 10 μM SB216763 for 30 min and then co-treated with 5 μM API-1 for an additional 2 h. **C**, The indicated cell lines were transfected with control (Ctrl) or GSK3α/β siRNA and 48 h later were exposed to DMSO or 5 μM API-1 for additional 4 h. **D**, H1299 cells were pre-treated with 10 μM SB216763 (SB) for 30 min and then co-treated with 5 μM API-1 for additional 4 h. The cells were then washed with PBS 3 times, refed with fresh medium containing 10 μg/ml CHX and cultured for the indicated times. **E**, The indicated plasmids were transfected into 293 T cells. After 48 h, the cells were treated with 5 μM API-1 for an additional 4 h. After the aforementioned treatments, the cells were harvested for preparation of whole-cell protein lysates and subsequent Western blotting. Protein levels were quantified with NIH Image J software and were normalized to actin. The results were plotted as the relative Mcl-1 levels compared to those at the time 0 of CHX treatment **(D)**. LE, longer exposure.

It is known that GSK3-mediated phosphorylation of Mcl-1 at S159 can trigger proteasomal degradation of Mcl-1 [8-11]. We therefore detected Mcl-1 phosphorylation in cells exposed to API-1. In API-1 sensitive H1299 and H522 cells, we detected a rapid and robust increase of p-Mcl-1 (S159/T163) early at 30 min post API-1 treatment followed by reduction of total levels of Mcl-1. In contrast, we detected a weak and late increase of p-Mcl-1 in API-1 insensitive H226 and H1792 cells (Figure 5A). Therefore, it is clear that API-1 increases Mcl-1 phosphorylation at S159, particularly in API-1 sensitive cell lines. We next determined whether API-1 induces a GSK3-dependent Mcl-1 reduction. The presence of the chemical GSK3 inhibitor SB216763 inhibited Mcl-1 phosphorylation and reduction induced by API-1 in both H1299 and H522 cell lines (Figure 5B). In agreement, genetic suppression of GSK3 with GSK3 siRNA clearly rescued Mcl-1 reduction induced by API-1 (Figure 5C). Moreover, inhibition of GSK3 with SB216763 reversed the effect of API-1 on shortening the half-life of Mcl-1 or on decreasing the stability of Mcl-1 (Figure 5D). Together, it is apparent that API-1 induces a GSK3-dependent Mcl-1 reduction or degradation. To further demonstrate the requirement of GSK3-mediated S159 phosphorylation in API-1-induced Mcl-1 degradation, we compared the effects of API-1 on reducing the levels of ectopically expressed wild-type and mutant Mcl-1, in which serine 159 (S159) was changed into alanine (S159A). As presented in Figure 5E, API-1 effectively decreased the levels of wild-type Mcl-1, but did not alter the levels of Mcl-1 (S159A). Hence, it is clear that the phosphorylation of S159 in Mcl-1 is an essential step for API-1-induced Mcl-1 degradation.

### API-1 induces GSK3-dependent apoptosis

**Figure 6:**
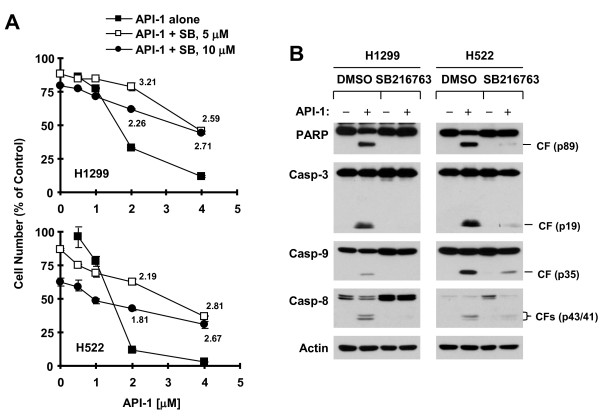
**Inhibition of GSK3 with SB216763 antagonizes the effects of API-1 on decreasing cell survival (A) and inducing caspase cleavage (B). A**, The indicated cell lines were exposed to different concentrations of API-1 alone, SB216763 alone and the combination of API-1 and SB216763. After 3 days, cell numbers were estimated with the SRB assay. Data are means ± SDs of four replicate determinations. The numbers in the graphs are CIs for the indicated combinations. **B**, The given cell lines were pre-treated with 10 μM SB216763 for 30 min and then co-treated with 5 μM API-1 for 24 h and then harvested for preparation of whole-cell protein lysates and subsequent Western blotting. CF, cleaved form.

If Mcl-1 reduction is a key event that mediates induction of apoptosis, we anticipated that blockage of GSK3-dependent Mcl-1 reduction will impair its ability to cause apoptosis. Hence, we further determined the impact of GSK3 inhibition on the ability of API-1 to induce apoptosis. By comparing the effects of API-1 on decreasing cell survival and inducing cleavage of caspases in the absence and presence of SB216763, we observed that co-treatment of cells with the API-1 and SB216763 combination was much weaker than API-1 alone in decreasing cell survival (Figure 6A) and in inducing cleavage of caspases including caspase-8, caspase-9, caspase-3 and PARP (Figure 6B) in both H1299 and H522 cells. The CIs for most combinations were > 1, suggesting antagonistic effects (Figure 6A). Thus, these data support the conclusion that API-1 also induces GSK3-dependent apoptosis.

### The SKP1-Cul1-F-box protein (SCF) E3 ligases, β-TrCP and FBXW7, are involved in mediating Mcl-1 degradation induced by API-1

**Figure 7:**
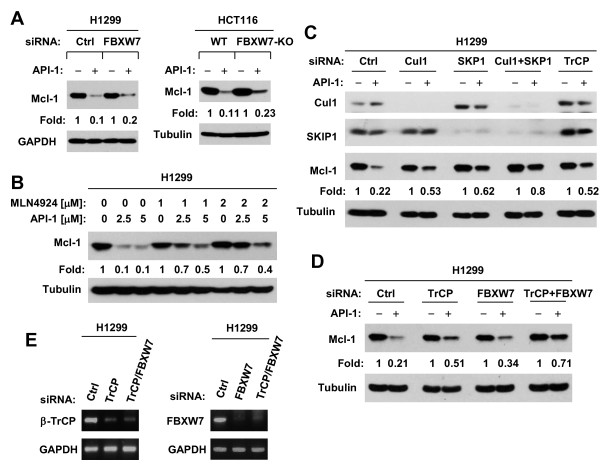
**Both the SCF E3 ligases, β-TrCP and FBXW7, are involved in mediating API-1-induced Mcl-1 degradation. A**, H1299 cells were transfected with control (Ctrl) or FBXW7 siRNA and 48 h later were exposed to DMSO or 5 μM API-1 for an additional 4 h. Moreover, WT and FBXW7-KO HCT116 cell lines were treated with 5 μM for 4 h. **B**, H1299 cells were pre-treated with the indicated concentrations of MLN4924 and then co-treated with the given concentrations of API-1 for an additional 4 h. **C** and **D**, H1299 cells were transfected with the given siRNAs for 48 h and then exposed to 5 μM API-1 for another 4 h. After these treatments, the cells were harvested for preparation of whole-cell protein lysates and subsequent Western blotting. Protein levels were quantified with NIH Image J software and were normalized to actin. **E**, H1299 cells were transfected with the given siRNAs and after 48 h were harvested for extraction of total RNA. RT-PCR was then conducted to detect the expression of the indicated mRNAs.

Finally, we investigated which E3 ubiquitin ligase is responsible for the GSK3-dependent proteasomal degradation of Mcl-1 induced by API-1. Given the recent studies on the critical role of FBXW7 in mediating GSK3-dependent degradation of Mcl-1 [10,11], we first determined whether this E3 ligase is involved in API-1-induced Mcl-1 degradation. Using the validated FBXW7 siRNA in our previous study [13], we detected slightly less reduction of Mcl-1 in FBXW7 siRNA-transfected cells than in control siRNA-transfected cells (Figure 7A). We further compared the effects of API-1 on Mcl-1 reduction between WT and FBXW7-KO HCT116 cells and generated similar results (Figure 7A) as we observed in the above knockdown experiment. These data collectively suggest that FBXW7 is only partly involved in mediating GSK3-dependent degradation of Mcl-1 by API-1, and that additional E3 ligases(s) are also involved in this process.

β-TrCP was previously suggested to mediate GSK3-dependent degradation of Mcl-1 [8]. We next determined whether this E3 ligase contributes to GSK3-dependent Mcl-1 degradation induced by API-1. Because both FBXW7 and β-TrCP are SCF E3 ligases and neddylation of Cul1 is required for the activity of SCF E3 ligases [25], we determined whether API-1 induces Mcl-1 degradation through a SCF E3 ligase-dependent mechanism. Inhibition of neddylation with MLN4924 impaired the ability of API-1 to decrease Mcl-1 levels (Figure 7B). In agreement, disruption of the SCF complex by knocking down of SKP1, Cul1 or both substantially rescued Mcl-1 reduction caused by API-1 (Figure 7C). These data clearly indicate the involvement of SCF E3 ligases in mediating API-1-induced Mcl-1 degradation. Following these experiments, we determined whether β-TrCP is involved in this process. As shown in Figure 7C, knockdown of β-TrCP substantially attenuated the ability of API-1 to decrease Mcl-1 levels, as did knockdown of Cul1 or SKP1, indicating that β-TrCP is indeed involved in mediating Mcl-1 degradation induced by API-1. Compared to the effects of β-TrCP or FBXW7 knockdown alone, co-knockdown of both β-TrCP and FBXW7 enhanced the rescuing effect on Mcl-1 reduction induced by API-1 (Figure 7D). The knockdown efficiencies of β-TrCP siRNA, FBXW7 siRNA or their combination were evaluated with RT-PCR (Figure 7E). Collectively, we suggest that both β-TrCP and FBXW7 are responsible for GSK3-dependent degradation of Mcl-1 induced by API-1.

## Discussion

Mcl-1 is a well-known Bcl-2 family protein that negatively regulates apoptosis by binding and sequestering the pro-apoptotic proteins such as Bax, Bak, Noxa, and Bim [7]. In this study, we found that API-1 rapidly and potently decreased Mcl-1 levels in NSCLC cell lines sensitive to API-1, but only minimally in API-1-insenstive cell lines. Moreover, enforced expression of ectopic Mcl-1 substantially protected NSCLC cells from undergoing apoptosis induced by API-1. These results clearly demonstrated that Mcl-1 reduction is an essential event for API-1 to induce apoptosis. In this study, API-1 also reduced the levels of survivin, another important anti-apoptotic protein that acts downstream of Mcl-1 as an endogenous inhibitor of caspases (e.g., caspase-9) [26]. However, we failed to demonstrate its essential role in mediating induction of apoptosis by API-1 because API-1 decreased survivin levels in NSCLC cells regardless of their sensitivities to API-1 and was equally effective in killing both NSCLC cells carrying a vector control and those expressing ectopic survivin. Hence the finding of Mc1 reduction as a critical mechanism accounting for API-1-induced apoptosis is novel. The current study focuses on demonstrating the role of Mcl-1 suppression in API-1-induced apoptosis. However, this does not exclude other targets or mechanisms such as Bcl-1 reduction described in this study (Figure 1) and c-FLIP degradation reported previously [2] that may account for API-1-induced apoptosis, particularly in a given cancer cell line. It is more likely that the effect of API-1 on induction of apoptosis is an outcome of the combination of multiple mechanisms including Mcl-1 reduction.

We noted that enforced expression of ectopic Mcl-1 blocked cleavage of not only caspase-9, but also caspase-8. It is very likely that caspase-8 activation caused by API-1 is secondary to activation of the intrinsic apoptotic pathway because caspase-8 can be activated by caspase-9 or caspase-3 [27,28]. However, we currently cannot rule out the possibility that caspase-8 activation is caused by Mcl-1 suppression if Mcl-1 has an uncovered role in direct suppression of the extrinsic apoptotic pathway.

We previously reported that API-1 effectively enhances TRAIL-induced apoptosis in human NSCLC cells involving induction of c-FLIP degradation [2]. In this study, we found that enforced expression of ectopic Mcl-1 protected NSCLC cells from induction of apoptosis by the combination of API-1 and TRAIL. Hence, it is apparent that Mcl-1 reduction is also an important mechanism by which API-1 augments TRAIL-induced apoptosis.

Mcl-1 is known to undergo GSK3-dependent proteasomal degradation [7,23]. As an Akt inhibitor, it was plausible to speculate that API-1 reduces Mcl-1 levels through activating GSK3 followed by enhancing GSK3-dependent Mcl-1 degradation. In this study, API-1 did not alter Mcl-1 mRNA levels, suggesting that API-1-induced Mcl-1 reduction is likely to be a posttranscriptional event. Indeed, inhibition of proteasome with MG132 rescued Mcl-1 reduction induced by API-1. Moreover, API-1 decreased Mcl-1 stability. Hence, it is clear that API-1 induces proteasomal degradation of Mcl-1, leading to Mcl-1 reduction. We observed that decreased Mcl-1 levels were accompanied with an early and robust increase in Mcl-1 phosphorylation at S159/T163, primarily in in those NSCLC cell lines sensitive to API-1. Importantly, suppression of GSK3 inhibited Mcl-1 phosphorylation and reduction or degradation induced by API-1. In agreement, API-1 lost its activity on inducing degradation of mutant Mcl-1 (S159A), in which S159 could not be phosphorylated. Collectively, we conclude that API-1 decreases Mcl-1 levels through facilitating GSK3-dependent proteasomal degradation of Mcl-1.

In our study, we found that inhibition of GSK3 (e.g., with SB216763) antagonized the effects of API-1 on decreasing the survival of NSCLC cells and on inducing cleavage of caspases and PARP. Thus, it is clear that API-1 induces GSK3-dependent apoptosis as well. This reinforces the critical role of GSK3-dependent Mcl-1 reduction in mediating apoptosis induced by API-1. This finding also implies that, to prevent potential antagonism, API-1 should not be used in combination with any agents that may inhibit GSK3 activity in the treatment of cancer.

Although FBXW7 has been recently suggested to be a key E3 ligase that mediates GSK3-dependent Mcl-1 degradation [10,11], we found that knockdown or knockout of FBXW7 provided only limited protective effect against Mcl-1 reduction induced by API-1. However, this process does require activation of SCF E3 ligases since the inhibition of SCF complex formation by knockdown of Cul1, SKP1 or both drastically impaired the ability of API-1 to decrease Mcl-1 levels. Hence, FBXW7 may not be the major E3 ligase responsible for API-1-induced GSK3-dependent proteasomal degradation of Mcl-1, and additional SCF E3 ligase(s) may be involved in mediating GSK3-dependent degradation of Mcl-1 induced by API-1. Indeed, our further studies have shown that β-TrCP, another SCF E3 ligase involved in mediating GSK3-dependent degradation of Mcl-1 as suggested previously [8], also contributes to API-1-induced Mcl-1 degradation since knockdown of β-TrCP provided a more drastic effect than FBXW7 knockdown on rescuing Mcl-1 reduction induced by API-1. Moreover, we found that co-knockdown of both β-TrCP and FBXW7 exhibited much more potent effects than knockdown of either single gene in preventing Mcl-1 reduction induced by API-1. Therefore, we believe that both β-TrCP and FBXW7 are involved in mediating GSK3-dependent Mcl-1 degradation induced by API-1. Our findings clearly suggest that two E3 ubiquitin ligases can cooperate to regulate the degradation of one protein.

## Conclusions

The current study has demonstrated that API-1 decreases Mcl-1 levels through facilitating GSK3-dependent, β-TrCP and FBXW7-mediated protein degradation. By reducing Mcl-1 levels, API-1 is able to induce apoptosis and sensitize cancer cells to TRAIL-induced apoptosis.

## Abbreviations

API-1: 4-amino-5,8-dihydro-5-oxo-8-β-D-ribofuranosyl-pyrido[2,3-*d*]pyrimidine-6-carboxamide; TRAIL: Tumor necrosis factor-related apoptosis-inducing ligand; NSCLC: Non-small cell lung cancer; GSK3: Glycogen synthase kinase 3; β-TrCP: β-Transducin repeats-containing protein; FBXW7: F-box/WD repeat-containing protein 7; WT: Wild-type; GAPDH: Glyceraldehyde 3-phosphate dehydrogenase; CHX: Cycloheximide; SRB: Sulforhodamine B; CI: Combination index; siRNA: Small interfering RNA; Cul1: Cullin1; RT-PCR: Reverse transcription-PCR; SCF: SKP1-cullin1-F-box protein.

## Competing interests

All authors declare that they have no competing interests.

## Authors’ contributions

HR, JK, BG and PY designed and conducted experiments and data analysis. XD, MC and FRK participated in discussion of the data. XD provided some critical reagents. MC and SYS participated in experimental design, coordination, data analysis and draft of the manuscript. All authors read and approved the final manuscript.

## Authors’ information

H Ren, J Koo and B Guan share the first authorship.
